# Sex Difference in the Association between Physical Activity and All-Cause Mortality in Ambulatory Patients with Chronic Kidney Disease

**DOI:** 10.3390/ijerph18073698

**Published:** 2021-04-01

**Authors:** Stig Molsted, Inge Eidemak, Mette Aadahl

**Affiliations:** 1 Department of Clinical Research, Nordsjællands Hospital, 3400 Hillerød, Denmark; 2 Department of Oncology, Section of Palliative Medicine, 2100 Copenhagen, Denmark; inge.eidemak@regionh.dk; 3 Center for Clinical Research and Prevention, Frederiksberg and Bispebjerg Hospital, 2000 Fredeiksberg, Denmark; mette.aadahl@regionh.dk; 4 Institute of Public Health, Faculty of Health and Medical Sciences, University of Copenhagen, 1455 Copenhagen, Denmark

**Keywords:** chronic kidney failure, cox proportional hazards models, dialysis, physical activity, survival analyses

## Abstract

(1) Background: The purpose of this article was to investigate the association between self-reported physical activity (PA) and all-cause mortality in ambulatory patients with chronic kidney disease (CKD), stage 4–5 including maintenance dialysis. (2) Methods: Ambulatory patients with CKD (eGFR < 30 mL/min/1.73 m^2^) with conservative treatment or chronic dialysis were included. PA was assessed using the Saltin–Grimby Physical Activity Level Scale. A Cox proportional hazards regression model––adjusted for age, sex, plasma–albumin, body mass index, socioeconomic status, and treatment––was applied. (3) Results: Participants (*n* = 374) were followed 39 ± 15 months from entry to death or censoring. Throughout the study period of 39 months, 156 deaths (42%) were registered. Regarding physical activity, 128 (34%) of the participants were inactive, 212 (57%) were moderately active, and 34 (9%) were highly or vigorously active. Moderate PA was associated with a decreased mortality risk in women (*n* = 150) compared to inactivity (HR 0.27 (0.15; 0.51), *p* < 0.001), whereas a high/vigorous level of PA was not significantly associated with mortality risk compared to inactivity. In men (*n* = 224), the associations between PA levels and mortality risk were not significant. (4) Conclusions: Moderate PA was associated with reduced all-cause mortality in ambulatory women with stage 4–5 CKD with or without maintenance dialysis treatment. Physical activity was not significantly associated with mortality in men.

## 1. Introduction

Physical activity (PA) is associated with a reduced risk of all-cause mortality in the general population [1,2], and even a modest amount of regular PA below the recommended 150 min of moderate-intensity PA per week [3] markedly reduces the risk of premature mortality [4]. Previous studies have also documented an association between PA and mortality in patients with chronic kidney disease (CKD) who undergo maintenance dialysis. In patients who had recently started hemodialysis (HD) or peritoneal dialysis (PD), an elevated mortality risk was observed in those who reported that they had never or almost never performed exercise training compared to those who exercised as little as “less than once a week” [5]. Likewise another study in patients who recently started HD or PD showed a dose-response relationship with decreased risk of all-cause mortality with increasing levels of self-reported PA [6]. A positive dose-response relationship between accelerometer-measured habitual PA and survival has also been demonstrated in patients undergoing HD [7].

Although the aforementioned studies indicate that patients undergoing HD or PD may reduce their risk of premature mortality through regular PA, rehabilitation programs with PA interventions to patients with CKD are not offered as standard procedures in most countries [8]. In addition, there is limited knowledge of the role of PA in patients with stage 4–5 CKD with conservative treatment. This knowledge is necessary as a basis for developing future evidence-based recommendations and guidelines on PA in clinical practice for patients with CKD. Whilst it may be relevant in general to offer all patients exercise training supervision or instruction, this study included ambulatory patients only to focus on those who could perform physical activity without severe limitations.

The aim of this study was to investigate whether the risk of all-cause mortality differed with self-reported PA levels in patients with stage 4–5 CKD including maintenance dialysis.

## 2. Material and Methods

This prospective cohort study was initialized in April 2015 in the three departments of nephrology and dialysis centers in the capital region of Denmark. The participants were included if they were ≥18 years of age, had been treated with maintenance HD or PD ≥three months or had been conservatively treated for stage 4–5 CKD (eGFR <30 mL/min/1.73 m^2^), had not been admitted to a hospital during the previous month, and were able to walk ≥50 m (a cane was allowed as a maximum aid). Exclusion criteria were dementia, severe psychiatric disorder, and an inability to read and understand Danish.

A total of 716 patients were invited. Of these, 539 chose to participate and were included. In the original cohort [9] some participants had missing data on PA as a result of uncomplete replies on the questionnaire, and body mass index (BMI) was not reported in all patient records. As the BMI is reported to be associated with the mortality risk in patients with CKD [10,11,12], this study included participants only with a registered BMI and a reported PA level. Thus, 374 participants were included in the present study.

### 2.1. Main Exposure: Physical Activity

The self-reported level of PA was determined using the Saltin–Grimby Physical Activity Level Scale [13,14]. This questionnaire has been frequently used in studies of chronic patient populations and in general population samples; in this case, the nationwide Danish Health and Morbidity Survey [15]. The tool was used to assess PA during leisure time with the question:

If we look at the past year, what would you say best describes your leisure time activities? The four response categories were:

(1) High intensity sports several times during a week
(2) Minimum four hours of exercise training weekly
(3) Minimum four hours of moderate activity as walking or cycling weekly
(4) Read, watch television or other sedentary activities

As only two participants reported the highest level of activity (High-intensity sports several times during a week), the two highest PA levels (1 and 2) were collapsed into one level. Thus, the participants’ levels of PA were categorized into inactive, moderate active, and highly/vigorously active.

### 2.2. Co-Variates

Potential confounders were selected a priori based on previous findings in the literature: socioeconomic status was defined as the participants’ level of education using a question from the Danish Health and Morbidity Survey and reported on the questionnaire by the participants and summarized as: low (<10 years of education), medium (10–12 years), and high (≥13 years). The BMI, obtained in the patient records, was calculated using height and weight (weight/height^2^) and categorised as (<25 kg/m^2^ vs. ≥25 kg/m^2^) because a higher BMI is associated with a reduced mortality rate in patients undergoing dialysis [10,11,12]. Plasma (P)-albumin values have also been associated with mortality [16], and the values were collected from patient records. Age, sex and smoking data were reported on the questionnaire.

To characterize the cohort, a question from the Danish Health and Morbidity Survey was used to obtain data for marital status defined as being married or having a permanent partner (“yes/no”). Likewise, blood (B)-hemoglobin, P-phosphate, eGFR, and renal disease were collected from patient records.

### 2.3. Outcome

Data for all-cause mortality were provided by The Danish Health Data Authority on June 2, 2020, from The Danish Civil Registration System. The register is updated every workday. The data were delivered according to the rules of The Danish Health Data Authority and the Data Protection Agency.

### 2.4. Ethics

The participants gave their informed consent. The study was approved by the local ethical committee (H-6-2014-103) and the Data Protection Agency (P-2020-197). The study was conducted in accordance with The Helsinki Declaration.

### 2.5. Statistical Analyses

Differences between physically active and inactive participants were tested using a One-way ANOVA test with a Tukey’s honestly significant difference post hoc test for continuous variables, and a χ^2^ test for categorical variables.

A multivariable Cox proportional hazards regression model was used to investigate the association between PA level and mortality. Time (months) was measured as the time from baseline to death or censuring. Necessary assumptions on linearity and proportional hazards were tested using polynomials of age and P-albumin. Analyses were adjusted for the covariates of age, P-albumin, BMI (<25 vs. ≥25 kg/m^2^), socioeconomic status (three levels), and treatment group (HD/PD vs. conservative treatment). Interactions between PA and sex, PA and BMI, and PA and treatment groups were tested. The mortality risk throughout the study period was also investigated using an unadjusted Kaplan–Meier survival plot. Statistical analyses were performed using the IBM SPSS 25 program. Data were presented as number (percentage), mean ± standard deviation (SD) or hazard ration (HR) (95% confidence interval (CI)). Results were considered significant (2-tailed) for *p* < 0.05.

## 3. Results

The participants were followed 39 ± 15 months from entry to death or to the last follow-up time. There were 156 deaths (42%) throughout the study period. Physical inactivity was reported by 128 (34%) participants, 212 (57%) were moderate active, and 34 (9%) were highly/vigorously active. The participants’ characteristics are presented in Table 1. There were no differences among the three groups’ continuous variables in the initial or the post hoc analyses. There were differences between women and men in age (62 ± 14 vs. 66 ± 13 years, respectively, *p* = 0.003), hemoglobin (7.2 ± 1.0 vs. 7.4 ± 0.9 mmol/L, respectively, *p* = 0.045), renal disease (diabetes 11 vs. 15%; hypertension 5 vs. 13%; polycystic 8 vs. 5%; glomerulonephritis 18 vs. 10%; pyelonephritis 1 vs. 0%; interstitial nephritis 3 vs. 1%; obstructive uropathy 1 vs. 5%; nephrosclerosis 11 vs. 10%; unknown 12 vs. 16%; and not reported 29 vs. 25%, respectively, *p* = 0.012), and mortality (34 vs. 47%, respectively, *p* = 0.013).

**Figure 1:**
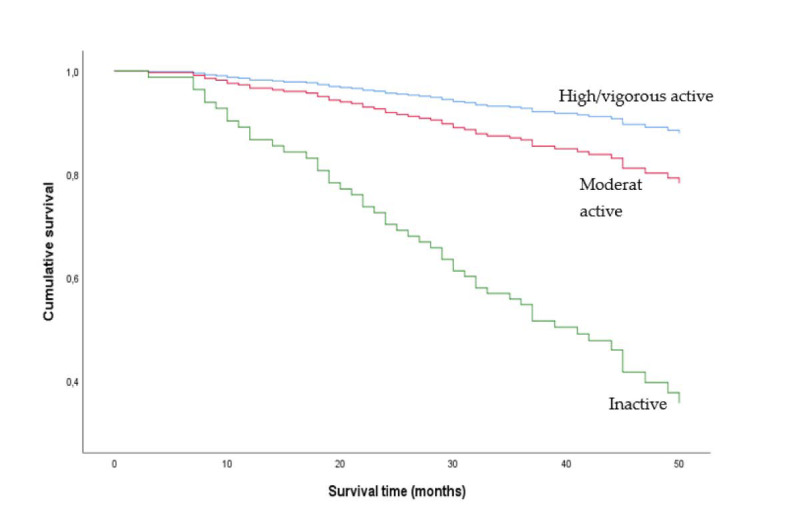
Unadjusted Kaplan-Meier plot of women who were moderately physically active, highly/vigorously active and inactive. Physical activity levels were determined using the Saltin–Grimby Physical Activity Level Scale.

**Figure 2:**
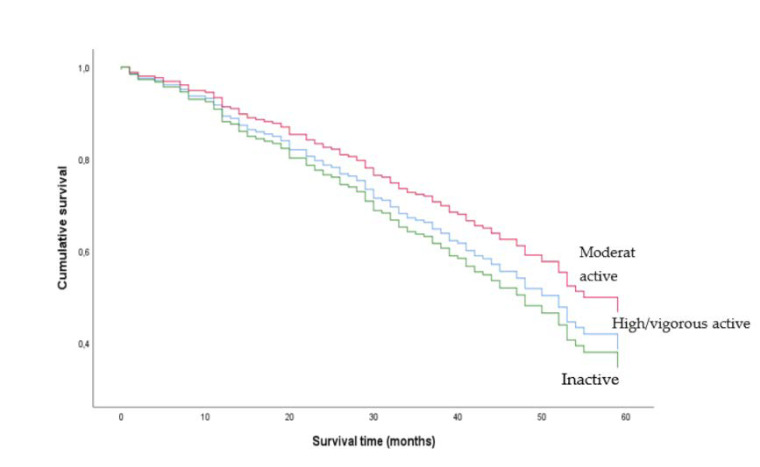
Unadjusted Kaplan-Meier plot of men who were moderately physically active, highly/vigorously active and inactive. Physical activity levels were determined using the Saltin–Grimby Physical Activity Level Scale.

Survival without adjustments among physically inactive, moderately active, and highly/vigorously active women and men are presented in Figure 1 and Figure 2, respectively. The adjusted Cox proportional hazards regression model is presented in Table 2. There was a significant interaction between PA and sex (HR 0.28 (0.15; 0.53), *p* < 0.001), and the analyses were stratified by sex.

Moderate PA was associated with a decreased mortality risk compared to inactivity during the study period in women (HR 0.27 (0.15; 0.51), *p* < 0.001), whereas the association between high/vigorous PA vs. inactivity and mortality was not significant. In men, the associations between moderate and high/vigorous PA vs. inactivity and mortality during the study period were not significant (Table 2). There were no interactions between PA and BMI, and PA and treatment groups.

The included participants from the original cohort had a higher mortality rate compared to the excluded participants (42% vs. 32%, respectively, *p* = 0.036), a higher prevalence of patients undergoing dialysis (74% vs. 41%, respectively, *p* < 0.001), and a younger age (65 ± 13 vs. 68 ± 13 years, respectively, *p* = 0.005). The sex distribution in the included (40% women) and excluded participants (34% women) was not different (*p* = 0.174).

## 4. Discussion

In the present study, an increased risk of all-cause mortality was found in physically inactive women with stage 4–5 CKD including maintenance dialysis treatment compared to women who were moderately physically active. The level of PA was not significantly associated with the mortality rate in male patients.

This study found different associations between PA levels and mortality in women and men. Previous studies on CKD populations did not find the same sex difference as presented in this study. However, to the best of our knowledge, only Johansen and colleagues investigated interactions with sex and did not report any sex-specific effect of PA on mortality in patients new to dialysis [6]. Whilst the percentage of inactive participants among men was somewhat lower than among women, the estimates in women and men were clearly different, and the results were not likely to be affected by a different distribution of PA level between women and men. However, men may overestimate their level of PA more than women [17]. Thus, the association between PA and mortality may have been biased to a greater extend in men than in women by the nature of the self-reported data. In addition, the raw PA data on men showed a pattern in the Kaplan–Meier plot that was not anticipated. The curves in the figure indicated that participants with moderate PA had an elevated survival compared to high/vigorous PA, a result that was not in agreement with the dose-response relationship shown in the female participants.

Whilst moderate PA vs. inactivity was associated with mortality in women, the association between high/vigorous PA vs. inactivity was not significantly associated with mortality in women. The explanation for the latter association without significance may be the result of a relatively low number of women with high/vigorous levels of PA, and a relatively wide 95% CI.

The impact of a moderate to high level of PA on mortality has been suggested to be the result of the positive effects on classic cardiovascular risk factors. Indeed, previous studies on patients undergoing HD have suggested that PA had a positive effect on, for example, blood pressure [18], glucose tolerance [19], CRP [20], heart rate variability [21], and visceral fat [22]. As cardiovascular mortality is frequent in patients with CKD, the aforementioned mechanisms may explain the association between PA and a reduced mortality in this and previous studies.

Even though there is an increasing body of evidence of the more positive effects of PA in patients with CKD, physical inactivity remains a problem for this patient group [23]. This was also true in a recent analysis of the cohort from the present study, where 61% of the ambulatory patients undergoing HD were physically active vs. 80% in an age- and sex-matched population [9]. Ambulatory patients were included to investigate associations in a cohort where a physically active lifestyle was possible. Two previous studies also focused on the potential effect of PA in ambulatory patients undergoing dialysis and found positive associations between PA and mortality risk. O’Hare and colleagues found that exercise training or PA during leisure time “never or almost never” vs. “less than once a week or more” reduced the mortality risk [5], and Matsuzawa and colleagues found that more time spend on PA was associated with a reduced mortality risk [7]. The same pattern was found in other studies with different stratifications of participant levels of PA [6], and when regular exercise training was investigated [24,25,26]. However, an interaction between PA and sex needs further investigation. 

The clinical implications of the findings of the present study include that PA should be recommended to reduce the risk of premature mortality in patients with CKD, especially in women. As PA not only may reduce the mortality risk but also the aforementioned cardiovascular risk factors. These data underline the need for guidelines and rehabilitation programs in clinical practice. Patients may need more intensive support and instruction in exercises to be motivated for an increased level of PA, and this could be delivered by nurses, medical doctors, physiotherapists and other health care workers.

Recommendations on PA for patients with CKD should include a brisk walk, which may not be understood as exercise training. The advice could also ideally be supported by exercise training programs at the dialysis centres. Communication to patients should also mention that PA has the potential to improve physical function [27], a parameter that may have a significant effect on patients’ motivation for PA. In order to elevate their level of PA in general, clinical departments may benefit from a shared strategy where all staff members support patients in their motivation for PA.

The present study may be limited by more factors, and residual confounding cannot be ruled out. Mortality in patients with CKD may be affected by several factors that were not included in this data collection or analysis. Smoking was not included in the analysis as this variable had a high number of missing data. Nor was diabetes included as a confounder as those data were often missing from the patient records. However, Johansen and colleagues found in their analysis that diabetes was not significantly associated with mortality [6]. Shimoda and colleagues also found it not be associated with mortality in an analysis of the impact of low functional mobility, muscle weakness and low serum albumin [28]. An important limitation was the missing data on the BMI that often were not reported in the patient records. The study should have been designed with collection of BMI data through the questionnaire at a minimum. An obvious limitation was that the PA level was self-reported. Self-reporting PA is well-known to be prone to substantial reporting bias, e.g., social desirability and recall bias [17]. In addition, the Saltin–Grimby Physical Activity Level Scale was not an ideal tool because it does not measure PA frequency. However, it was suggested to be a robust tool for measuring PA in surveys [29]. Finally, even though the study had a prospective design, the results may be affected by an insufficient adjustment for disease severity. However, disease severity is not defined by a single variable, and it was to some extent adjusted for by including P-albumin as a covariate, and by limiting the population to include ambulatory patients only.

In conclusion, a moderately physically active lifestyle was associated with a substantial reduction of risk of all-cause mortality in ambulatory women with stage 4–5 CKD with or without maintenance dialysis treatment. In men, physical activity was not significantly associated with the mortality rate. However, in clinical practice, physical activity should be recommended for all patients as it has positive effects. The differences or interaction between physical activity and sex should be tested in future studies on the association between physical activity level and mortality risk.

## Figures and Tables

**Table:** 

Number at Risk							
Months	0	10	20	30	40	50	60
Highly/vigorously active							
	9	9	9	9	7	3	
Moderately active							
	78	78	76	68	47	20	4
Inactive							
	63	57	47	35	19	9	3

**Table:** 

Number at Risk							
Months	0	10	20	30	40	50	60
Highly/vigorously active							
	25	25	22	19	14	8	2
Moderately active							
	134	126	115	100	68	29	4
Inactive							
	65	60	54	43	28	12	1

**Table 1:** Characteristics of the physically inactive, moderate active and highly/vigorously active participants (*n* = 374).

Variable	Physical Inactive (*n* = 128)	Moderate Active (*n* = 212)	High/Vigorous Active (*n* = 34)	*p*
Age (years)	66 ± 13	64 ± 13	62 ± 16	0.351
Sex				0.018
Women	63 (49%)	78 (37%)	9 (27%)	
Men	65 (51%)	134 (63%)	25 (74%)	
Marital status				0.219
Married/had a partner	68 (53%)	132 (63%)	21 (62%)	
No partner	60 (47%)	79 (37%)	13 (38%)	
Socioeconomic status				0.028
Low	31 (24%)	32 (15%)	11 (32%)	
Medium	62 (48%)	98 (46%)	11 (32%)	
High	35 (27%)	82 (39%)	12 (35%)	
Treatment				0.035
Hemodialysis	96 (75%)	124 (59%)	20 (59%)	
Peritoneal dialysis	8 (6%)	25 (12%)	3 (9%)	
Conservative	24 (19%)	63 (30%)	11 (32%)	
Dialysis vintage (months) ^a^	58 ± 51	45 ± 49	53 ± 40	0.258
Body mass index (kg/m^2^)	26.8 ± 6.4	26.2 ± 5.4	24.9 ± 3.8	0.195
Hemoglobin (mmol/L)	7.1 ± 0.9	7.4 ± 0.9	7.4 ± 0.8	0.063
Plasma albumin (g/L)	36 ± 5	36 ± 5	37 ± 4	0.721
Plasma phosphate (mmol/L)	1.6 ± 0.5	1.6 ± 0.5	1.5 ± 0.4	0.580
Bikarbonat (mmol/L)	23.8 ± 3.5	24.6 ± 3.0	24.5 ± 3.5	0.119
C-reactive protein (mg/L)	9 ± 20	9 ± 23	6 ± 7	0.761
eGFR (mL/min/1.73 m^2^) ^b^	19 ± 7	22 ± 8	22 ± 9	0.693
Renal disease				0.209
Diabetes	20 (16%)	26 (12%)	4 (12%)	
Hypertension	4 (3%)	28 (13%)	5 (15%)	
Polycystic	3 (2%)	17 (8%)	3 (9%)	
Glomerulonephritis	17 (13%)	26 (12%)	7 (21%)	
Pyelonephritis	0 (0%)	1 (1%)	0 (0%)	
Interstitial nephritis	3 (2%)	3 (1%)	0 (0%)	
Obstructive uropathy	4 (3%)	9 (4%)	1 (3%)	
Nephrosclerosis	17 (13%)	21 (10%)	2 (6%)	
Unknown	15 (12%)	32 (15%)	6 (18%)	
Not reported ^c^	45 (35%)	49 (23%)	6 (18%)	
Smoking				0.068
Yes, daily	24 (19%)	21 (10%)	3 (9%)	
Yes, not daily	6 (5%)	8 (4%)	0 (0%)	
No	74 (58%)	149 (70%)	23 (68%)	
Not reported ^c^	24 (19%)	34 (16%)	8 (24%)	
Died during follow up	69 (54%)	72 (34%)	15 (44%)	0.001
Hemodialysis	54 (49%)	48 (43%)	9 (8%)	0.035
Peritoneal dialysis	5 (31%)	10 (63%)	1 (6%)	0.495
Conservative treatment	10 (35%)	14 (48%)	5 (17%)	0.098

Data are presented as mean ± SD or n (%). ^a^ In participants undergoing dialysis, ^b^ in conservative treated participants, ^c^ not reported (was not included in the analysis of the difference between groups).

**Table 2:** Estimated hazard ratios from the Cox regression analyses of the association between mortality and the physical activity level determined using the Saltin–Grimby Physical Activity Level Scale in women and men. The analyses were adjusted for predetermined confounders.

Physical Activity	HR (95% CI)	*p*
Women		
Inactive	1	
Moderate active	0.27 (0.15; 0.51)	<0.001
High/vigorous active	0.19 (0.03; 1.46)	0.111
Men		
Inactive	1	
Moderate active	0.78 (0.49; 1.23)	0.284
High/vigorous active	1.13 (0.59; 2.18)	0.716

The analysis was adjusted for age, P-albumin, BMI (<25 vs. ≥25 kg/m^2^), treatment (dialysis vs. conservative), and education (three levels).

## Data Availability

The data presented in this study are available on request from the corresponding author. The data are not publicly available due to the Danish law.
